# Multiomics analysis investigating the impact of a high-fat diet in female Sprague–Dawley rats: alterations in plasma, intestinal metabolism, and microbial composition

**DOI:** 10.3389/fnut.2024.1359989

**Published:** 2024-04-05

**Authors:** Jiacheng Zhang, Binhong Hu, Xin Deng, Rong Sun, Rong Zhang, Kuo Chen, Wenzhi Guo

**Affiliations:** ^1^Department of Hepatobiliary, Pancreatic and Liver Transplantation Surgery, The First Affiliated Hospital of Zhengzhou University, Zhengzhou, China; ^2^Henan Key Laboratory of Digestive Organ Transplantation, Zhengzhou, China; ^3^Open and Key Laboratory of Hepatobiliary and Pancreatic Surgery and Digestive Organ Transplantation at Henan Universities, Zhengzhou, China; ^4^College of Chemistry and Life Sciences, Chengdu Normal University, Chengdu, China; ^5^School of Physical Science and Technology, ShanghaiTech University, Shanghai, China; ^6^Department of Breast Surgery, The First Affiliated Hospital of Zhengzhou University, Zhengzhou, China; ^7^Henan Research Centre for Organ Transplantation, Zhengzhou, China

**Keywords:** high fat diet, gut microbiota, metabolome, *Akkermansia*, bile acids

## Abstract

**Introduction:**

With improvements in living conditions, modern individuals exhibit a pronounced inclination towards a high-fat diet, largely because of its distinctive gustatory appeal. However, the association between high-fat diets and metabolic complications has largely been ignored, and metabolic diseases such as obesity and non-alcoholic fatty liver disease now constitute a major public health concern. Because high-fat diets increase the risk of metabolic diseases, a thorough investigation into the impact of high-fat diets on gut microbiota and metabolism is required.

**Methods:**

We utilize 16S rRNA sequencing and untargeted metabolomics analysis to demonstrate that SD rats fed a high-fat diet exhibited marked alterations in gut microbiota and plasma, intestinal metabolism.

**Results:**

Changes in gut microbiota included a decreased abundance at phylum level for Verrucomicrobiota, and a decreased abundance at genus level for *Akkermansia, Ralstonia, Bacteroides*, and *Faecalibacterium*. Additionally, significant changes were observed in both intestinal and plasma metabolite levels, including an upregulation of bile acid metabolism, an upregulation of glucose-lipid metabolism, and increased levels of metabolites such as norlithocholic acid, cholic acid, D-fructose, D-mannose, fructose lactate, and glycerophosphocholine. We also investigated the correlations between microbial communities and metabolites, revealing a significant negative correlation between *Akkermansia* bacteria and cholic acid.

**Discussion:**

Overall, our findings shed light on the relationship between symbiotic bacteria associated with high-fat diets and metabolic biomarkers, and they provide insights for identifying novel therapeutic approaches to mitigate disease risks associated with a high-fat diet.

## 1 Introduction

Long-term consumption of a high-fat diet (HFD) is a significant risk factor for a diverse range of public health diseases, including obesity, inflammatory bowel disease, and reproductive damage. HFD consumption has also been shown to gradually impair function in multiple organs (1–4). Other consequences include dyslipidemia and an alteration in lipid and plasma transaminase profiles (5). During the development of these lipid disorders, the body typically encounters adaptive barriers to nutrition and to the environment. These changes include modifications to molecular mechanisms associated with metabolic damage. For instance, HFD significantly promotes hepatic lipid droplet accumulation while modulating SIRT-1/AMPK pathways and influence gene expression related to lipid synthesis and degradation (5, 6).

Gut microbiota dysbiosis is also closely associated with long-term HFD consumption. Studies of the relationship between diet-related diseases and microbiota have progressively shifted from correlation studies to inquiries on causality and interactions (7–9). Because HFD modulates the composition of gut microbial communities, the integrity of the intestinal barrier is compromised (7, 10). Moreover, the resulting damage to the intestinal mucosa and increase in lipopolysaccharide (LPS) production stimulate systemic inflammation and metabolic disorders, which are factors in the development of obesity and type 2 diabetes (T2DM) (11, 12). In addition, fatty acids derived from microbes exacerbate diet-induced obesity and further compromise intestinal epithelial integrity (13). Disordered intestinal microbiota and its metabolites can promote inflammation, for example, the enrichment of arachidonic acid and lipopolysaccharide biosynthetic pathways found in the feces of young adults on a high-fat diet (14). Therefore, it is necessary to understand the mechanism of intestinal microbiota mediating HFD, and then design personalized dietary strategies by regulating intestinal microbiota (15, 16).

Understanding the impact of long-term HFD consumption on metabolism is crucial for elucidating the interaction between host metabolism and microbiota. In HFD-fed mice, supplementation of medium-chain and long-chain triglycerides (MLCT) improved energy utilization, enhanced regulation of glucose and lipid metabolism, and inhibited inflammation (17). HFD consumption is also known to activate fatty acid oxidation processes that mediate intestinal stemness and tumorigenicity (18). Bile acids (BAs) are synthesized endogenously in the liver, and they subsequently circulate to distal organs such as the intestine where they exert profound effects (19). These essential metabolic products play an important role in liver diseases. Gut microbiota also produce short-chain fatty acids and BAs that promote hepatic homeostasis through their interaction with mitochondria (20). Modulation of the BA signaling pathway along the “gut-liver axis” has emerged as a novel approach for ameliorating obesity and metabolic disorders. Supporting evidence for this approach comes from the observation that primary BAs and dietary fat intake supplementation can improve metabolic disorders (21). Notably, intestinal BA levels are significantly elevated in diet-induced obese mice (22). Despite these advances, a comprehensive understanding of the dynamic characteristics induced by long-term HFD consumption is lacking, and further elucidation of HFD-induced changes may facilitate the identification of effective intervention strategies.

In the present study, we aim to investigate the impacts of long-term HFD consumption on the interaction between microbiota and metabolites using 16S rRNA sequencing and untargeted metabolomics approaches. Our objectives include the detection of potential symbiotic bacteria, the identification of biomarkers associated with HFD consumption, and an elucidation of the underlying mechanisms linking gut microbiota and host metabolism.

## 2 Materials and methods

### 2.1 Animal models

All animal experiments were approved by the Ethics Office of the First Affiliated Hospital of Zhengzhou University, China (no. 2023-KY-0658), and conducted in accordance with the ARRIVE guidelines and the National Institutes of Health Guidelines for the Care and Use of Laboratory Animals. The 24 female Sprague Dawley (SD) rats (3–4 weeks of age) (23) were purchased from Yuchi Experimental Animal Co. Ltd., China. The rats were housed in a controlled environment (temperature, 25 ± 3°C; humidity, 75 ± 5%), under a 12-h light/dark cycle, and provided with sufficient food and water. The rats were separately randomly divided into two groups (*n* = 12): CON (normal diet) and HFD (60% fat, 20% protein, and 20% carbohydrates). These experimental groups were then maintained for a duration of 60 days. For the sample sequencing, we employed a method of combining two microbiota samples and conducted a pooled treatment of four samples within each metabolic group to ensure biological replicability.

### 2.2 Blood index detection

At the end of the experimental period, 0.5 mL of blood was collected from SD rat through the tail vein (*n* = 6). All blood samples were then incubated for 15 min, and the serum was isolated by centrifugation at 2000 ×*g* for 10 min. Serum levels of estradiol (E2), triglyceride (TG), total cholesterol (TC), low-density lipoprotein cholesterol (LDL-C), high-density lipoprotein cholesterol (HDL-C), interleukin (IL)-6, and tumor necrosis factor (TNF)-α were analyzed using specific enzyme-linked immunosorbent assay (ELISA) kits according to the manufacturer’s instructions. The blood samples were paired and subjected to testing using a kit.

### 2.3 Oral glucose tolerance test

After the 60-day intervention, rats were given a glucose load (2 g/kg) by oral gavage. Blood glucose levels were measured using a glucose oxidase reagent strip 30, 60, 90, and 120 min before and after glucose administration (24).

### 2.4 16S rRNA sequencing

Colorectal contents of SD female rats were collected from 6 per group for 16S rRNA sequencing. FLASH (V1.2.11) (25) was used to assemble the sample reads to obtain the Raw Tags. These were subjected to quality control using fastp. The Clean Tags obtained after quality control were compared with the Silva database, and chimeras were detected and removed using Vsearch (25). DADA2 (by default) or deblur in QIIME2 (Version QIIME2-202006) (26, 27) were used for denoising, and sequences with relative abundance less than 5% in the total microbial profile were filtered out (28) to obtain the final amplicon sequence variants (ASVs) and feature table. The species in each sample were annotated using the Silva database through QIIME2 software.

### 2.5 Metabolomics analysis

Plasma samples (100 μL) and colorectal contents (60 mg) from SD female rats were further processed for metabolomics analysis. First, 300 μL of the protein precipitation agent methanol-acetonitrile (V:V = 2:1, containing L-2-chlorophenylalanine 2 μg/mL) and 600 μL of methanol–water (V:V = 4:1, containing L-2-chlorophenylalanine 4 μg/mL) was added to each sample of plasma and colorectal contents, respectively. Next, the mixture was vortexed and ultrasonically extracted. The resulting solution was filtered through a 0.22 μm organic phase pinhole filter for LC–MS analysis. LC was performed on an ACQUITY UPLC column (150 mm × 2.1 mm, 1.8 μm) using water (containing 0.1% formic acid) as solvent A and acetonitrile as solvent B (flow rate, 0.35 mL/min; injection volume, 5 μL). The positive and negative ion scanning modes were both employed for relative quantitative analysis of samples using Progenesis QI v2.3 software.

### 2.6 Statistical analysis

All data were presented as mean ± standard deviation. GraphPad Prism 8.0 was used for all statistical analyses. A two-tailed *t*-test was used to compare differences between two groups, and one-way analysis of variance (ANOVA) was used to compare multiple groups. Differences with a *p* < 0.05 were considered statistically significant. Correlation between genus-level flora and inflammatory factors, TG, TC, LDL-C, HDL-C was assessed using Spearman’s rank correlation coefficient.

## 3 Results

### 3.1 Consumption of a high-fat diet induces weight gain, upregulates inflammatory factors, and disrupts lipid metabolism in female Sprague–Dawley rats

**Figure 1:**
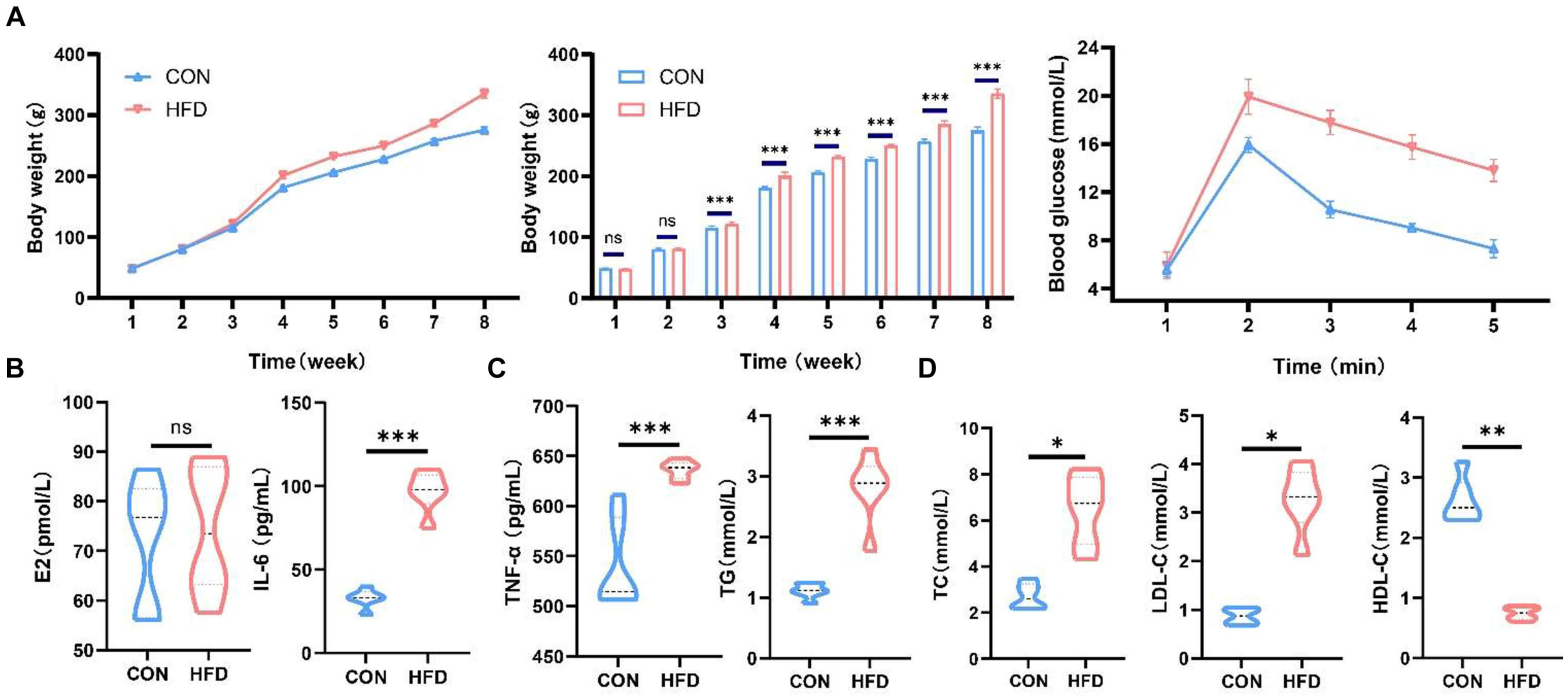
High-fat diet induces weight gain and dyslipidemia in female SD rats. **(A)** Changes in body weight and oral glucose tolerance test in rats on normal or high-fat diets. **(B)** Serum estradiol levels in female rats. **(C)** Serum IL-6 and TNF-α levels in female rats. **(D)** Serum TG, TC, HDL-C and LDL-C levels in female rats. ^*^p < 0.05, ^**^p < 0.01, ^***^p < 0.001.

HFD consumption induced an increase in body weight in SD rats (Figure 1A). Oral glucose tolerance test demonstrating changes in blood glucose concentration in each group (Figure 1A). We investigated the changes in estradiol levels in female rats and found no difference in estradiol levels between normal female rats and rats in the HFD group (Figure 1B). Moreover, consumption of an HFD by SD female rats resulted in elevated inflammatory factor levels and disrupted lipid metabolism (Figures 1C,D). While levels of TG, TC, and LDL-C were elevated, HDL-C levels were markedly decreased.

### 3.2 High-fat diets lead to changes in the composition of the gut microbiota

**Figure 2:**
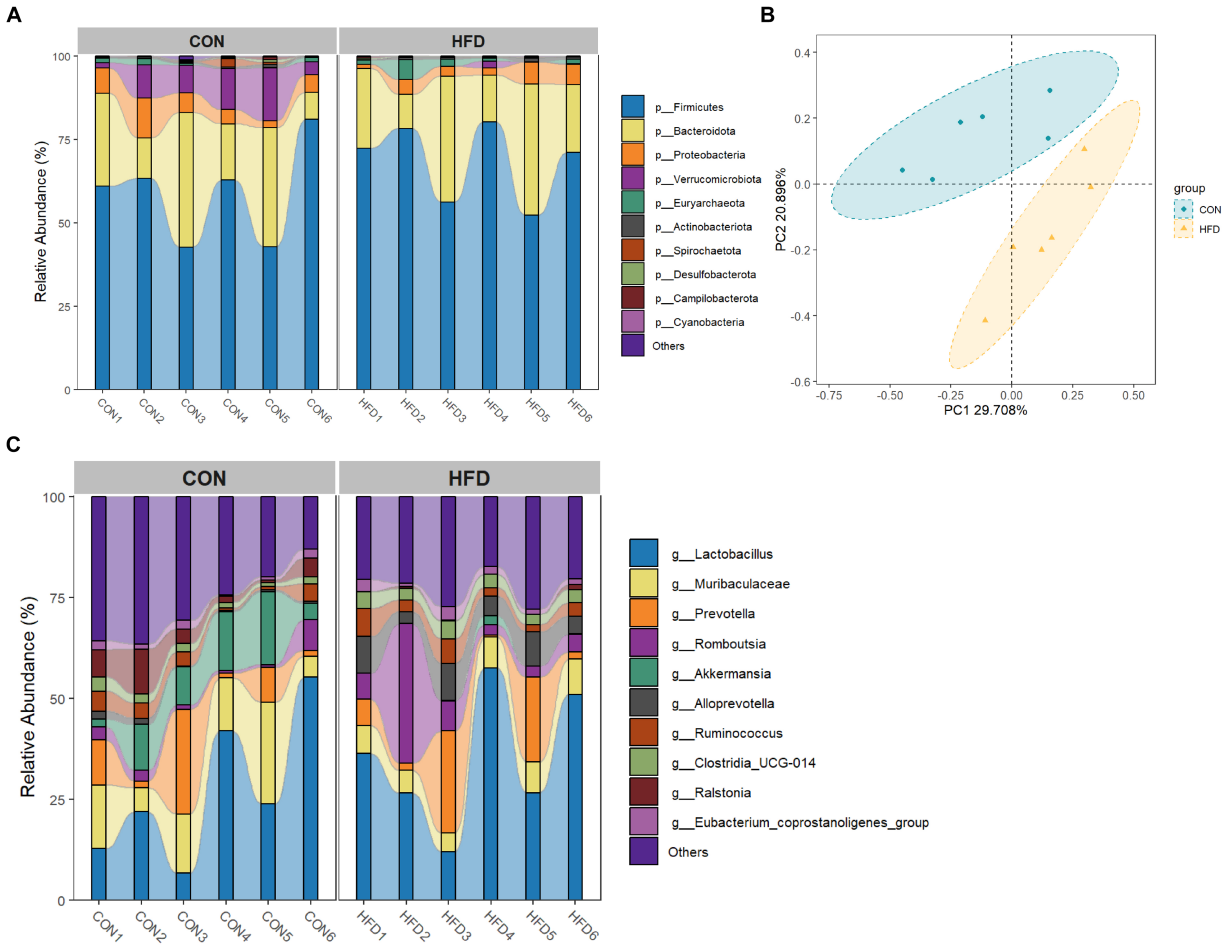
High-fat diet-induced changes in the gut microbiota of rats. **(A)** Phylum level bar chart. **(B)** Genus level bar chart. **(C)** PCoA analysis based on Bray Curtis dissimilarity index.

After data merging and quality control of the raw sequencing data, we successfully obtained a total of 59,442–68,217 high-quality sequence reads of the 16S rRNA gene from 12 samples of intestinal content. Analysis of these reads reveals no statistically significant difference in the α-diversity of gut microbiota between rats fed a normal diet and rats fed an HFD. The top ten bacterial taxa are presented in a species bar chart at both phylum level (Figure 2A) and genus level (Figure 2B). At the phylum level, Firmicutes, Bacteroidota, Proteobacteria, Verrucomicrobiota, and Euryarchaeota were predominant. At the genus level, *Lactobacillus*, *Muribaculaceae*, *Prevotella*, *Romboutsia*, and *Akkermansia* were predominant. Principal co-ordinates analysis (PCoA) was employed to compare microbial community distribution among the different groups (Figure 2C), revealing a noticeable disparity in gut microbiota composition between rats fed on normal diet and rats fed on HFD.

### 3.3 Rats fed a high fat diet exhibit a different microbial composition

**Figure 3:**
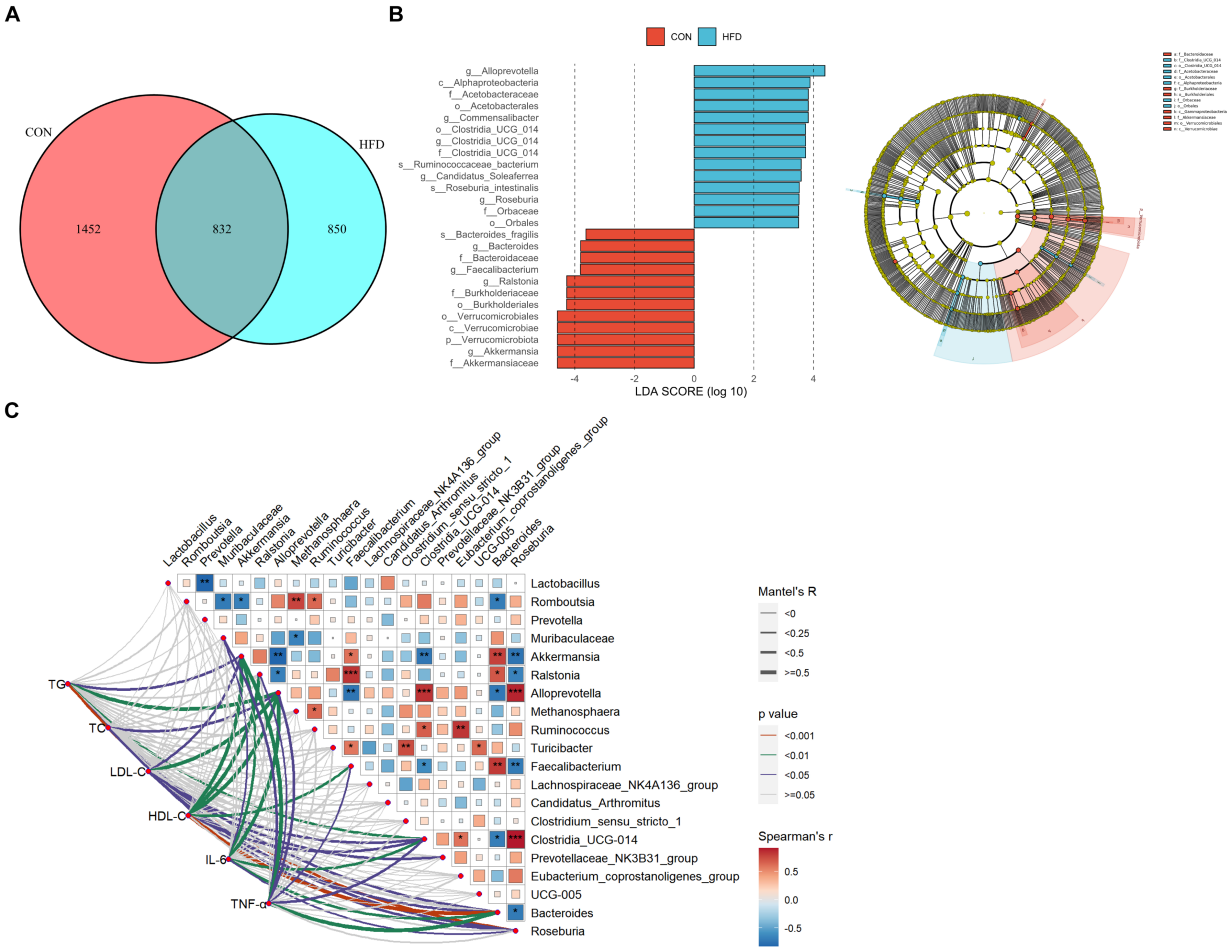
The gut microbiota of rats fed a high-fat diet exhibited significant differences compared to the gut microbiota of rats fed a normal diet. **(A)** Venn chart. **(B)** LEfSe analysis and evolutionary branch chart. Intergroup comparisons were conducted using the Wilcoxon rank sum test (LDA, 3.5). **(C)** Heatmap of mantel test correlation.

A Venn plot showing shared and unique ASVs obtained from rats fed a normal diet and rats fed a HFD is presented in Figure 3A. Linear discriminant analysis Effect Size (LEfSe) analysis indicated that at the phylum level, Verrucomicrobiota dominated in the CON group. At the genus level, *Akkermansia*, *Ralstonia*, *Bacteroides*, and *Faecalibacterium* were dominant in the CON group, whereas *Alloprevotella*, *Commensalibacter*, *Clostridia_UCG_014*, and *Candidatus_Soleaferrea* were dominant in the HFD group (Figure 3B). The above findings demonstrate marked alterations in gut microbiota composition in rats fed a HFD. In addition, the associations of intestinal flora with the expression of inflammatory factors and dyslipidemia were calculated based on spearman correlation analysis (Figure 3C).

### 3.4 A high-fat diet elicits modifications in the composition of intestinal contents and plasma metabolites

**Figure 4:**
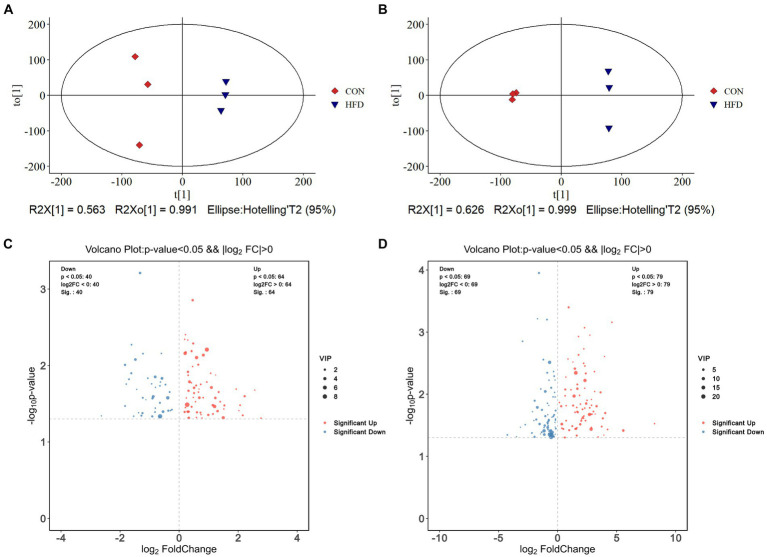
High-fat diet-induced alterations in colorectal contents metabolites and plasma metabolites. **(A)** OPLS-DA score plot of colorectal contents metabolites. **(B)** OPLS-DA score plot of plasma metabolites. **(C)** Volcano plot demonstrating changes in colorectal contents metabolites. **(D)** Volcano plot demonstrating changes in plasma metabolites.

OPLS score charts for intestinal contents (Figure 4A) and plasma samples (Figure 4B) illustrate the changes in metabolite distribution between the CON group and HFD group. In total, 104 different metabolites were identified in intestinal content samples (R2Y = 0.991, Q2 = 0.606) with 64 up-regulated and 40 down-regulated metabolites detected in positive and negative ion modes, respectively. In addition, 148 differential metabolites were identified in plasma samples (R2Y = 0.999, Q2 = 0.91) with 79 up-regulated and 69 down-regulated in positive and negative ion modes, respectively. To facilitate their identification, the screened differential metabolites (*p* < 0.05 and VIP > 1.0) were visualized in Volcano plots (Figures 4C,D).

**Figure 5:**
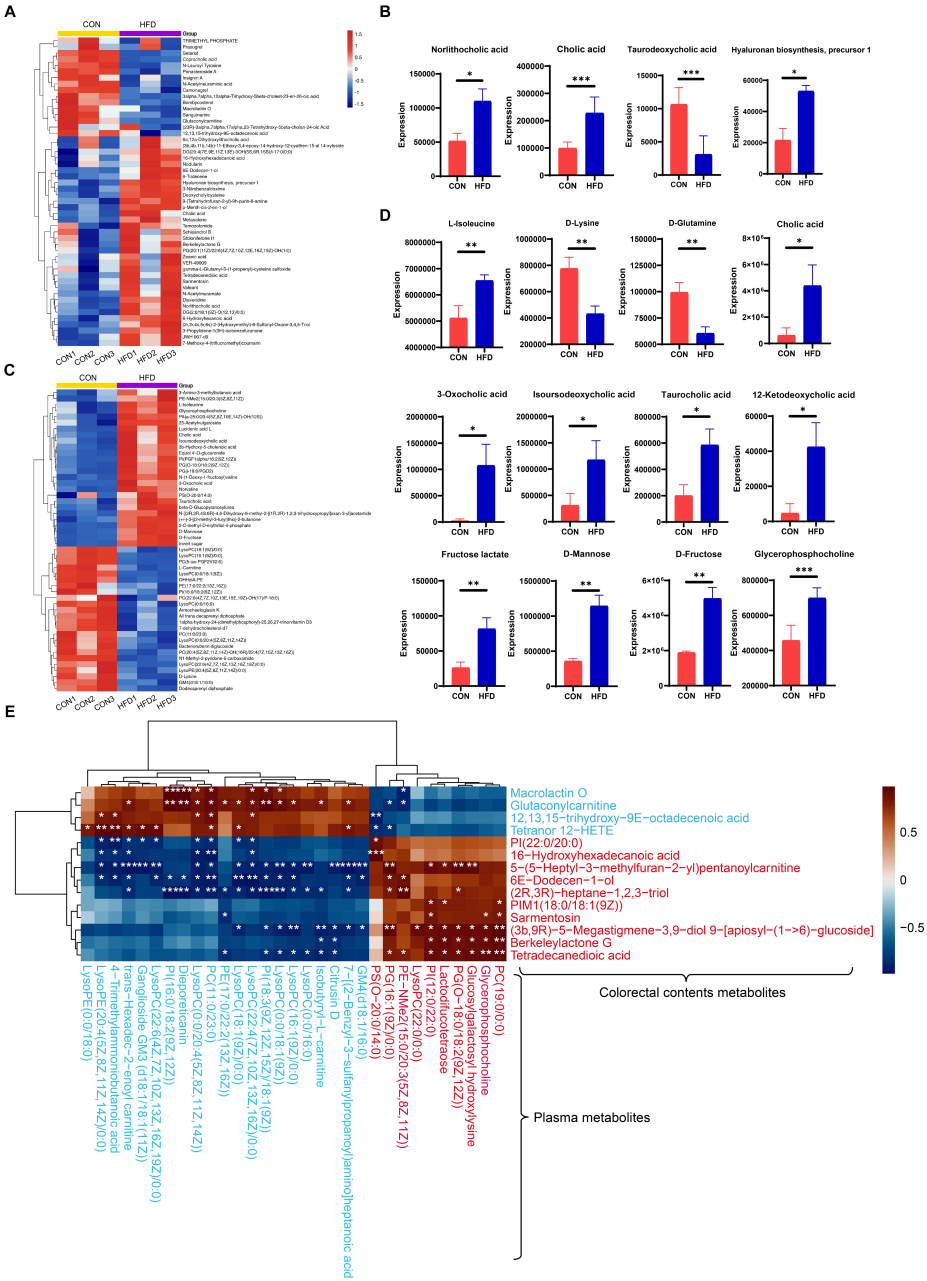
Analysis of alterations in gut content metabolites and plasma metabolites. **(A)** Heatmap displaying the top 50 metabolites present in the gut. **(B)** Identification of key metabolites within the gut. **(C)** Heatmap displaying the top 50 metabolites present in plasma. **(D)** Identification of key metabolites within plasma. **(E)** Correlation heatmap depicting differential metabolites associated with lipid metabolism. The blue text denotes downregulation of these metabolites in the HFD group compared to the control, while the red font indicates upregulation. ^*^p < 0.05, ^**^p < 0.01, ^***^p < 0.001.

In addition, heat maps were used to identify differences in the TOP50 metabolites between the CON and HFD groups (for intestinal contents, Figure 5A; for plasma samples, Figure 5C). The key metabolites were subsequently identified from these differential metabolites. Significant differences in the metabolites between experimental groups (*p* < 0.05) were confirmed using a two-tailed t-test (for intestinal contents, Figure 5B; for plasma samples, Figure 5D). For the intestinal contents, the key metabolites included norlithocholic acid, cholic acid, taurodeoxycholic acid, and hyaluronan biosynthesis precursor-1 (Figure 5B). For the plasma samples, the upregulated key metabolites included L-isoleucine, cholic acid, isoursodeoxycholic acid, taurocholic acid, 12-Ketodeoxycholic acid, D-Fructose, D-mannose, fructose lactate, and glycerophosphocholine, and the down-regulated key metabolites were D-lysine and D-glutamine (Figure 5D). Pearson correlation analysis showed the correlation of colorectal contents with plasma lipid metabolism-related metabolites (Figure 5E).

### 3.5 High-fat diets induce alterations in metabolic pathways within the intestinal tract and plasma

**Figure 6:**
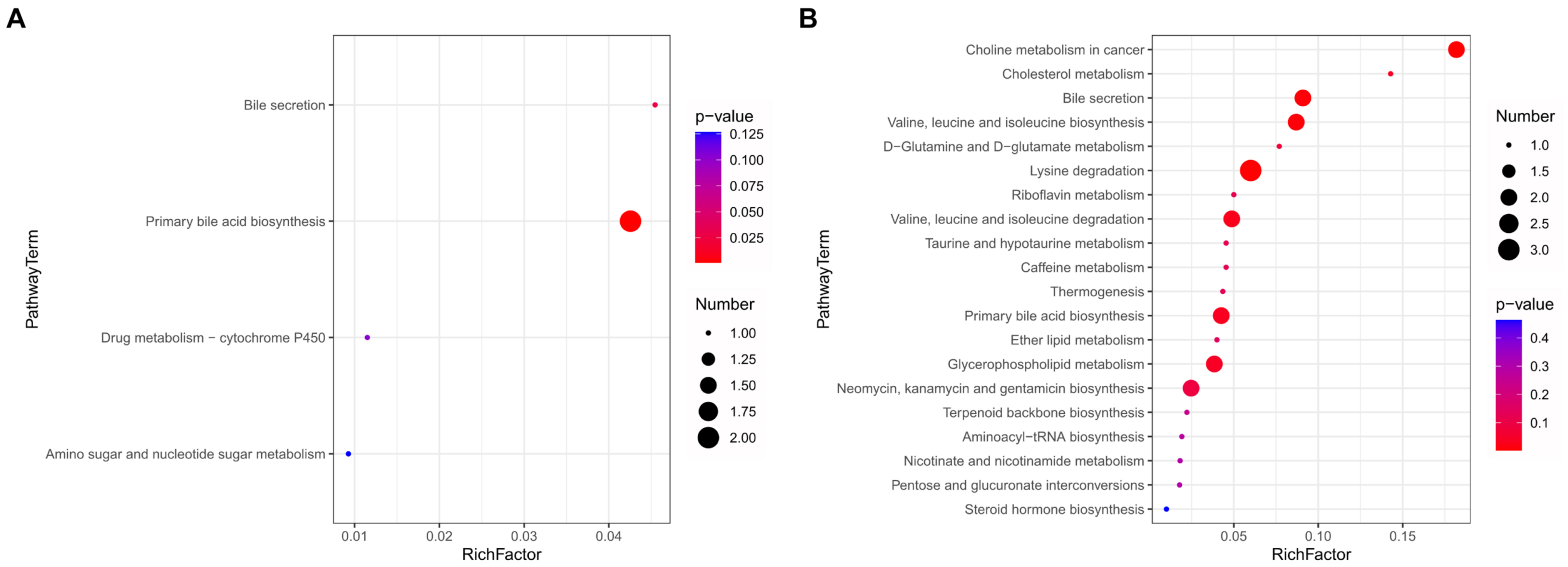
High-fat diet-induced changes in top 20 metabolic pathways. **(A)** Enrichment map of metabolic pathways for intestinal contents. **(B)** Enrichment map of metabolic pathways for plasma samples.

To further elucidate the impact of HFD consumption on metabolic pathways, we performed KEGG analyses. For the intestinal contents, significantly enriched upregulated pathways were predominantly associated with “bile secretion” and “primary bile acid biosynthesis” (Figure 6A). For the plasma samples, the significantly enriched upregulated pathways included “bile secretion,” “valine, leucine and isoleucine biosynthesis and degradation,” and “primary bile acid biosynthesis,” while the significantly enriched downregulated pathways included “Lysine degradation” and “D-Glutamine and D-glutamate metabolism” (Figure 6B). Notable alterations in “choline metabolism in cancer” were also observed. Collectively, these findings provide evidence that HFD consumption is associated with perturbations in amino acid metabolism, energy metabolism, and bile acid metabolism.

### 3.6 Correlation between gut microbiota and metabolites

**Figure 7:**
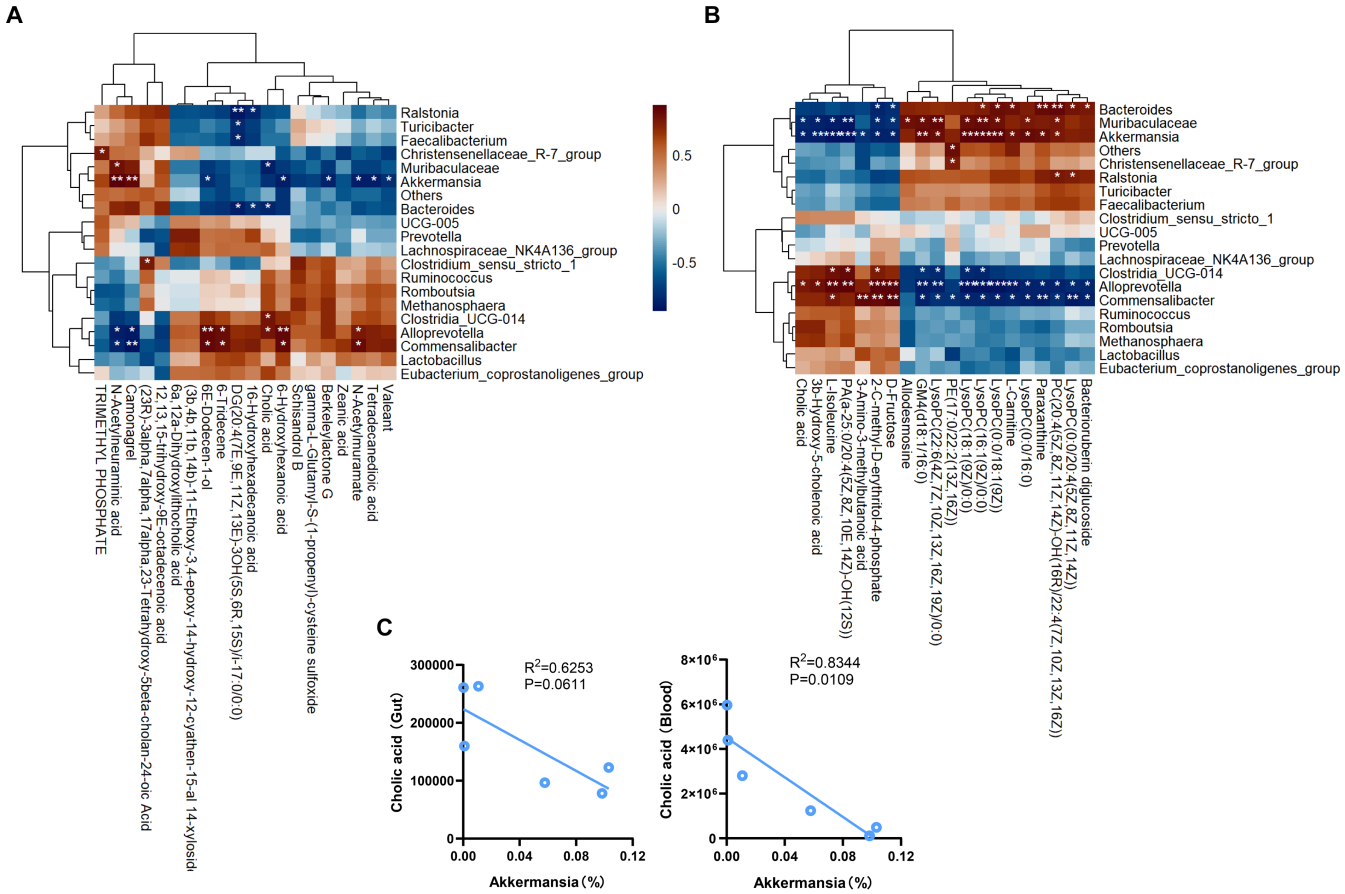
Pearson correlation analyses between gut microbiota and various metabolic products. **(A)** The relationship between gut content metabolism and gut microbial community. **(B)** The relationship between plasma metabolism and gut microbial community. **(C)** Correlation analysis. ^*^p < 0.05, ^**^p < 0.01, ^***^p < 0.001.

Pearson correlation analysis revealed negative correlations between *Akkermansia* and the intestinal contents metabolites cholic acid, berkeleylactone G, tetradecanedioic acid, N-Acetylmuramate, and valeant. Conversely, our analysis also revealed positive correlations between *Akkermansia* and the intestinal contents metabolites N-Acetylneuraminic acid and camonagrel (Figure 7A). In addition, Pearson correlation analysis revealed negative correlations between *Akkermansia* and the plasma metabolites cholic acid, 3b-hydroxy-5-cholenoic acid, 3-amino-3-methylbutanoic acid, D-fructose and L-isoleucine. Conversely, our analysis also revealed positive correlations between *Akkermansia* and the plasma metabolites GM4 (d18:1/16:0) and LysoPC (0:0/18:1(9Z)) (Figure 7B). Analysis of the correlation between *Akkermansia* and bile acids (Figure 7C), showed a negative correlation.

## 4 Discussion

Animal models fed HFD are commonly utilized to investigate different metabolic diseases, e.g., non-alcoholic fatty liver disease, hypercholesterolemia, and obesity (5, 29, 30), and these diseases are closely associated with dysbiosis of gut microbiota. Diet is known to be a crucial factor influencing alterations of gut microbiota (31). An unhealthy diet (such as long-term HFD consumption) not only leads to unfavorable metabolic outcomes, but it also induces changes in microbiota composition, which consequently increases intestinal permeability, allowing toxic bacterial metabolites to enter the circulatory system, ultimately promoting systemic inflammation (32). In the present study, we observed an increased secretion of pro-inflammatory factors in the blood of HFD-fed rats (in comparison with control rats), along with alterations in the composition of their gut microbiota. Specifically, at the phylum level, there was a decrease in the abundance of microorganisms other than Verrucomicrobiota within the HFD group. At the genus level, we noted a reduction in *Akkermansia*, *Ralstonia*, *Bacteroides*, and *Faecalibacterium* abundance. Conversely, *Alloprevotella*, *Commensalibacter*, *Clostridia_UCG_014*, and *Candidatus_Soleaferrea* exhibited an increase in abundance. It is noteworthy that individuals with gastrointestinal-related diseases or abnormalities often exhibit decreases in the levels of *Faecalibacterium* and *A. muciniphila* — two species primarily responsible for butyric acid production — and this is contrary to what is typically observed in a healthy human gut (33, 34). *A. muciniphila* also influences mucin production, and its abundance in the flora is reduced in cases of obesity and mild inflammation (35). Indeed, supplementation with *A. muciniphila* alone can loss weight and improve metabolic disorders (35). Furthermore, *A. muciniphila* converts mucin into beneficial by-products, contributing to the regulation of intestinal homeostasis and the maintenance of intestinal barrier integrity (36). In conclusion, the decrease in *A. muciniphila* levels observed in HFD may be closely associated with intestinal damage and metabolic disorders. The abundance levels of Bacteroides, which secrete bile acid hydrolase (37), are also down-regulated in rats fed an HFD. Some similar observations were previously reported in study on T2DM and long-term HFD (22, 33).

Under normal dietary conditions, there exists a robust temporal and spatial correlation among metabolites, enabling metabolic communication and coordination across multiple tissues and organs. However, long-term HFD consumption disrupts these intricate metabolic networks (38). In the present study, we employed non-targeted metabolomics approaches to evaluate the impact of an HFD on plasma and intestinal metabolic profiles. We observed changes in lipid metabolism-related metabolites, and Pearson analysis demonstrated a correlation between lipid metabolism-related metabolites in colorectal contents and plasma. Among the plasma metabolites, two LysoPEs and seven LysoPCs were downregulated in the HFD group relative to control. Consistent with our study, significant reductions in plasma LysoPCs levels were also found in obese and T2DM patients (39). Importantly, in the group fed an HFD, we observed a significant elevation in cholic acid levels in both the plasma and within the gut. It is already well-established that HFD consumption induces an increase in BA concentrations within the liver, stool, and plasma (40). In samples of intestinal contents, we observed an HFD-induced increase in the levels of norlithocholic acid, taurodeoxycholic acid, hyaluronan biosynthesis precursor-1, and other metabolites. In plasma samples, we observed elevations in the levels of isoursodeoxycholic acid, taurocholic acid, and 12-ketode oxycholic acid. BAs are known to play a crucial role in regulating intestinal function (22, 41), and their concentrations are strongly influenced by dietary factors and alterations in gut microbiota (42). A reduction in BA levels can alleviate changes in gut microbiome induced by HFD and subsequently mitigate obesity phenotypes (22). Of the symbiotic gut bacteria influenced by HFD consumption, *A. muciniphila* appears to be particularly involved in bile acid-host metabolism. Previous studies have demonstrated that most bile salts (including cholic acid) inhibit the growth of *A. muciniphila* (43). Indeed, we observed a significant negative correlation between *A. muciniphila* and cholic acid content. Furthermore, *A. muciniphila* has the potential to modulate BA production, leading to an inhibition of systemic inflammation, a reduction in blood glucose levels, and an improvement in glucose homeostasis (44, 45). Consequently, we postulate that a liver-BA-gut microbiome metabolic axis exists within the body, and that HFD-induced changes result in substantial alterations in both BA levels and microbiome composition, ultimately contributing to metabolic disorders. Therefore, a combined intervention targeting *A. muciniphila* and BA holds promise as an innovative approach for mitigating disease risk associated with HFD.

Estradiol levels are closely associated with the development of disorders related to glucose and lipid metabolism, such as diabetes and obesity (46, 47). Some studies have reported a reduction in estradiol levels following a high-fat diet (23), while others have not observed significant differences in estradiol levels among female rats under an HFD, consistent with our findings (46, 48). This discrepancy may be attributed to variations in glycolipid metabolism; however, the precise relationship remains unclear. Given the crucial role of estradiol in regulating metabolism and body weight homeostasis (49), further investigations are warranted to elucidate the association between estradiol levels and a HFD.

Metabolic disorders resulting from excessive fat consumption increase an individual’s susceptibility to disease occurrence, particularly by disrupting glucose and lipid metabolism (50). Long-term HFD consumption impacts serum levels of total cholesterol, low-density LDL-C, and HDL-C (51). In our present study, we also report increased plasma concentrations of D-fructose, D-mannose, lactate fructose, and choline glycerophosphate after HFD consumption. Thus far, elevated levels of glycerophosphocholine have been regarded as an indicator of aberrant choline metabolism in cancer (52). In summary, our study demonstrates significant HFD-induced alterations in metabolite levels in SD rats, especially in the levels of metabolites within BA metabolism and glycolipid metabolism pathways. We observed alterations in both plasma and intestinal bile acid levels, alongside changes in glucolipid metabolism among plasma metabolites, suggesting that plasma metabolites may serve as more suitable indicators for assessing the nutritional status of organisms on a HFD. To date, metabolic patterns have not been utilized to predict the health effects and underlying mechanisms of HFDs. Therefore, further investigation is warranted to elucidate specific metabolic signatures associated with nutrient/ microbiome interactions for intervention strategies, diagnostic purposes, and a comprehensive understanding of the impact of nutrition on the quantitative composition of healthy gut microbiota.

## 5 Conclusion

Our research findings demonstrate that long-term HFD consumption induces dyslipidemia in rats, leading to perturbations in gut microbiota and metabolism. In particular, we observed a reduction in the abundance of *A. muciniphila* and an elevation in BA levels among rats receiving an HFD. Correlation analysis revealed an inverse association between *A. muciniphila* and BA levels, both in the intestines and in plasma. In summary, our study elucidates the underlying mechanisms responsible for the deleterious effects of an HFD on organisms, and it also identifies potentially impacted metabolic pathways, specifically “bile acid metabolism” and “glucose-lipid metabolism”.

## Data availability statement

Original datasets are available in a publicly accessible repository: The original contributions presented in the study are publicly available. This data can be found here: [https://www.ncbi.nlm.nih.gov/bioproject/?term=PRJNA1067806/PRJNA1067806].

## Ethics statement

The animal study was approved by the Ethics Office of the First Affiliated Hospital of Zhengzhou University, China (no. 2023-KY-0658). The study was conducted in accordance with the local legislation and institutional requirements.

## Author contributions

JZ: Conceptualization, Data curation, Formal analysis, Funding acquisition, Methodology, Software, Writing – original draft, Writing – review & editing. BH: Data curation, Software, Writing – original draft. XD: Data curation, Formal analysis, Methodology, Software, Writing – original draft. RS: Formal analysis, Methodology, Software, Writing – original draft. RZ: Data curation, Software, Writing – original draft. KC: Project administration, Writing – original draft. WG: Conceptualization, Supervision, Writing – original draft, Writing – review & editing.
